# Spinal Muscular Atrophy in Colombia: Nationwide Incidence, Demographic Distribution, and Healthcare Challenges

**DOI:** 10.7759/cureus.95409

**Published:** 2025-10-25

**Authors:** Milena Villamil-Osorio, Cristian Andres Correa-Arrieta, Sandra Milena Castellar Leones, Edna Julieth Bobadilla-Quesada, Silvia Maradei-Anaya

**Affiliations:** 1 Pulmonology, Fundación Hospital Pediátrico la Misericordia, Bogotá, COL; 2 Pulmonology, Biotecnología y Genetica SAS Biotecgen, Bogotá, COL; 3 Neurology, Centro de Investigación en Fisiatría y Electrodiagnóstico (CIFEL), Bogota, COL; 4 Neurology, Biotecnología y Genetica SAS Biotecgen, Bogotá, COL; 5 Physical Medicine and Rehabilitation, Universidad Nacional de Colombia, Bogota, COL; 6 Physical Medicine and Rehabilitation, Biotecnología y Genetica SAS Biotecgen, Bogotá, COL; 7 Neurology, Fundación Hospital Pediátrico la Misericordia, Bogotá, COL; 8 Neurology, Hospital Universitario San Ignacio, Bogotá, COL; 9 Medical Genetics, Biotecnología y Genetica SAS Biotecgen, Bogotá, COL; 10 Genetics, Fundación Hospital Pediátrico la Misericordia, Bogotá, COL

**Keywords:** colombia, epidemiology, health surveillance, incidence, spinal muscular atrophy

## Abstract

Background: Spinal muscular atrophy (SMA) is a genetic neuromuscular disorder marked by progressive motor neuron degeneration, leading to muscle weakness and atrophy. Although rare, SMA severely impacts morbidity and quality of life, especially through respiratory complications. Limited data on SMA in Colombia hampers healthcare planning, emphasizing the need for region-specific studies to guide resource allocation and policy development.

Methods: This descriptive, longitudinal study analyzed SMA incidence in Colombia from 2016 to 2024, using data from the National Public Health Surveillance System (SIVIGILA) and the Health Services Information System (RIPS). Incidence rates were calculated per 100,000 live births, focusing particularly on SMA type 1. Temporal trends and subgroup distributions (by gender and geographic region) were examined using descriptive statistics.

Results: SMA incidence showed an upward trend, peaking in 2019 at 8.43 per 100,000 live births before stabilizing. SMA type 1 cases, representing the most severe form, accounted for a significant proportion, highlighting the intensive healthcare demands associated with this subtype. Geographic disparities in incidence were observed, with higher rates in regions with greater healthcare access, suggesting variability in diagnostic and reporting practices across Colombia.

Conclusion: This study offers critical insights into SMA’s epidemiology in Colombia, underscoring the healthcare burden of SMA type 1 and regional disparities in case reporting. Findings support the need for improved SMA surveillance, targeted resources in high-incidence regions, and possible implementation of newborn screening to enhance early diagnosis and care. These actions are essential for aligning SMA management in Colombia with international standards and addressing healthcare inequities.

## Introduction

Spinal muscular atrophy (SMA) is a genetic neuromuscular disorder characterized by the progressive degeneration and loss of motor neurons in the spinal cord and brainstem, resulting in muscle weakness and atrophy. SMA is primarily caused by mutations in the SMN1 gene, which encodes the survival motor neuron (SMN) protein, essential for maintaining motor neurons. The disorder is categorized into four main types based on age at onset and motor function achievement: type 1 is the most severe, presenting in infancy, while type 4 has a milder adult onset [1,2]. Globally, SMA is considered a rare disorder, with an estimated prevalence of 1-2 per 100,000 individuals and an incidence of approximately 1 in 10,000 live births [3,4], data obtained especially from European cohorts [5]. Despite its rarity, SMA profoundly impacts morbidity and quality of life, particularly due to respiratory complications frequently associated with its progression [6,7].

Although SMA is a rare disease, it is a significant public health concern for resource-limited settings due to its high health care costs, particularly for the Infantile SMA type 1 [8-10]. Furthermore, SMA is a disabling disease that affects not only the patient's health-related quality of life (HRQOL) but also causes a great burden for caregivers, requiring special attention and vigilance [9,11,12]. The prevalence of SMA varies by region and is influenced by both genetic and demographic factors. Carrier frequencies worldwide range from 1 in 40 to 1 in 70, indicating a substantial potential for cases across diverse populations [8,13].

Prior studies highlight the importance of large-scale epidemiological research and surveillance systems in accurately estimating SMA’s burden. Countries like Japan, Canada, and Italy have leveraged cross-sectional methodologies and national health databases to monitor SMA incidence, prevalence, and clinical characteristics [14-16]. Epidemiologic data on SMA in the Latin American population are minimal, derived mainly from descriptive studies and case reports. Only Brazil has incidence data derived from pilot neonatal screening programs, the results of which are similar to those reported worldwide [17]. In Chile, the implementation of the national SMA surveillance program made it possible to create a model to systematically collect clinical, motor and genetic data on SMA patients and to obtain demographic data, geographic distribution, social characteristics and economic income in the families of SMA patients, thus enriching local data [16].

Detailed epidemiological data on SMA in Colombia are limited, with inadequate data on prevalence, demographic patterns, and regional variations. Colombia’s National Epidemiological Surveillance System (SIVIGILA) plays a pivotal role in monitoring rare diseases, including SMA, under the "rare disease event" classification. However, detailed insights on SMA epidemiology in Colombia are lacking, underscoring the need for dedicated studies [18,19]. Lack of comprehensive data limits understanding of SMA’s impact on Colombia’s healthcare system, where genetic disorders are frequently underreported due to limited surveillance infrastructure [9,20]. Addressing these data gaps is crucial for a comprehensive understanding of SMA in Colombia, which could support effective healthcare planning resource allocation, policy making and specialized program development [11].

Reliable, region-specific data on SMA prevalence and demographics are essential for creating health interventions tailored to Colombia’s specific healthcare needs. Additionally, accurate data can guide public health funding, enhance access to early diagnostic tools, and facilitate genetic counseling and SMA-specific therapies [18]. With advancements in SMA treatments, obtaining timely, region-specific data is increasingly relevant for addressing this rare disease’s burden within Colombia.

This study presents data on SMA incidence in Colombia, with a focus on SMA type 1, taking into account its severity as a neurogenetic urgency [21], derived from records within the Sistema Nacional de Vigilancia en Salud Pública (SIVIGILA) and the Sistema de Información de Prestaciones de Salud (RIPS). We also describe sociodemographic characteristics relevant to SMA patients in Colombia, such as gender distribution in SMA type 1, and information on SMA type 2 and 3 is also presented, and regional differences in the country. This study aims to address these knowledge gaps, offering insights to enhance SMA management and inform policy-making within Colombia’s healthcare system. The subsequent sections detail the study’s methodology and findings.

## Materials and methods

Study design

This study is a descriptive, longitudinal analysis aimed at calculating the incidence of SMA and characterizing the demographic distribution of SMA patients in Colombia. Data from January 1, 2016, to July 31, 2024, were gathered from the National Public Health Surveillance System (SIVIGILA), and data from January 1, 2015, to October 31, 2024, from the Health Services Information System (RIPS). This design facilitates the examination of SMA incidence and distribution trends over time, with a focus on both overall SMA cases and subtype 1 (SMA type 1) in pediatric populations.

Data sources

Data for this study were obtained from Colombia's two primary health information systems: the National Public Health Surveillance System (SIVIGILA) and the Health Services Information System (RIPS). SIVIGILA data, publicly available through national information systems, provided annual records of new SMA diagnoses, which were used for incidence calculations and year-over-year case counts. RIPS contributed additional demographic details, healthcare interactions, and clinical data for SMA patients, filtered by specific ICD-10 codes associated with SMA. The ICD-10 codes used to identify SMA cases included G120 (Spinal muscular atrophy type I - Werdnig-Hoffman), G10-G13 (Systemic atrophy affecting the central nervous system), G121 (Other hereditary spinal muscular atrophy), G128 (Other spinal muscular atrophy and related syndromes), and G129 (Spinal muscular atrophy, not otherwise specified).

The State controls the official SIVIGILA and RIPS databases. SIVIGILA maintains internal control of the information. Children entering must have a confirmed genetic diagnosis. The system uses automatic validations, coding rules, and event filters to ensure data quality and consistency, avoiding duplicates or incomplete data. The RIPS database includes health services, and records can only be created for patients who are already part of SIVIGILA, ensuring data reliability. The analysis was conducted according to the country's political-administrative division, which is divided by department.

Official live birth counts for each year from the Colombian National Administrative Department of Statistics (DANE) were incorporated to calculate SMA incidence rates.

Inclusion and exclusion criteria

The inclusion criteria encompassed all new SMA diagnoses recorded in SIVIGILA and RIPS during the study period. Cases were identified using the specified ICD-10 codes. Records that were incomplete or lacked essential variables, such as diagnosis type or demographic information, were excluded to maintain accuracy. To avoid duplicate counting, records were cross-referenced between SIVIGILA and RIPS, with duplicates removed.

Variables analyzed

This study focused on several key variables: (i) Demographic variables: Age, gender, and geographical region of residence were recorded to examine the demographic distribution of SMA cases; (ii) Clinical variables: SMA type, specifically identifying SMA type 1, and year of diagnosis were noted for all cases; (iii) Incidence calculation: Annual incidence rates were calculated per 100,000 live births. For each year, the number of new SMA cases was divided by the total number of live births and then multiplied by a coefficient of 100,000. Separate incidence rates were calculated for overall SMA and SMA type 1; (iv) Trends over time: Yearly trends in SMA incidence were analyzed, and subgroup distributions (by gender, geographical region, and SMA type) were assessed to identify temporal and demographic trends.

Statistical analysis

Descriptive statistics summarized demographic and clinical variables, providing a detailed profile of SMA patients. Frequency distributions were generated for each variable, and incidence rates for both overall SMA and SMA type 1 were calculated annually from 2016 to 2024 to evaluate trends over time. Subgroup analyses, including gender and regional breakdowns, provided further insights into the distribution of SMA cases. Data analysis and visualization were conducted using Microsoft Excel (Microsoft Corporation, Redmond, USA) and IBM SPSS Statistics for Windows, Version 25 (Released 2017; IBM Corp., Armonk, New York, United States).

Ethical considerations

This study was approved by an institutional ethics review board, in accordance with Colombian health research regulations. Patient confidentiality and data privacy were safeguarded by anonymizing all personal identifiers during data processing, ensuring compliance with ethical standards for research involving health information.

## Results

Incidence of SMA

Using data collected from the National Public Health Surveillance System (SIVIGILA) and the Health Services Information System (RIPS), the annual incidence of SMA and SMA type 1 was calculated based on the number of new cases each year and annual live birth data from the DANE. As shown in Table 1, SMA incidence steadily increased from 2016 to 2019, peaking in 2019 at 8.43 cases per 100,000 live births. The incidence of SMA type 1 also reached a high in 2019, at 3.28 per 100,000 live births. Although incidence rates fluctuated post-2019, they remained elevated compared to earlier years, suggesting that improved detection and reporting practices or an actual increase in cases may have influenced these trends, warranting further investigation.

**Table 1:** Annual incidence of spinal muscular atrophy (SMA) and SMA type 1 during the study period. SMA: Spinal Muscular Atrophy; SMA Type 1: Spinal Muscular Atrophy Type 1; Incidence: Cases per 100,000 Population; Live Births: Total Births in the Respective Year.

Year	New SMA Diagnoses	New SMA Type 1 Diagnoses	Live Births	SMA Incidence per 100,000 Population	SMA Type 1 Incidence per 100,000 Population
2016	1	1	647,521	0.15	0.15
2017	9	4	656,704	1.37	0.60
2018	16	10	649,115	2.46	1.54
2019	54	21	639,82	8.43	3.28
2020	35	12	629,402	5.56	1.90
2021	40	13	616,914	6.48	2.08
2022	39	9	569,311	6.85	1.58
2023	35	16	573,625	6.10	2.78

Trends in SMA cases reported

**Figure 1:**
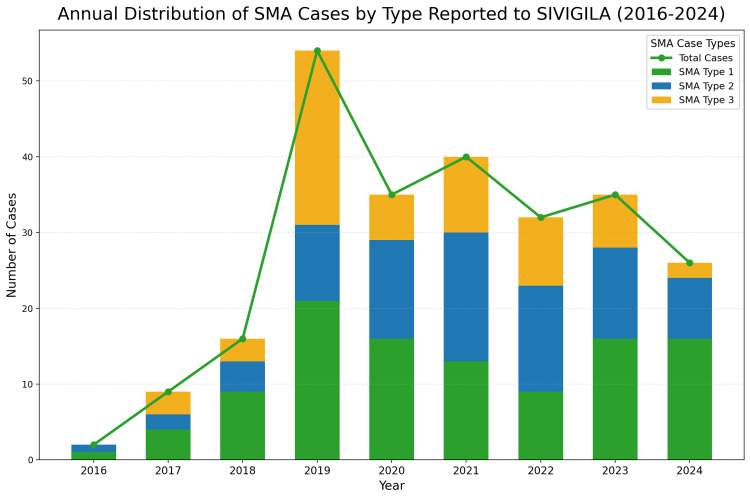
Annual distribution of SMA cases by type reported to SIVIGILA (2016-2024). This figure illustrates the annual number of spinal muscular atrophy (SMA) cases reported to SIVIGILA from 2016 to 2024, categorized by SMA type. The stacked bars represent the distribution of cases by type: SMA type 1 (green), SMA type 2 (blue), and SMA type 3 (yellow). The green line with data points depicts the total annual number of SMA cases reported. Notably, a peak in total SMA cases is observed in 2019, followed by a plateau and slight decrease in subsequent years. This trend may reflect improvements in diagnostic capabilities and healthcare access, as well as potential impacts from the COVID-19 pandemic on case reporting and healthcare priorities.

The annual distribution of SMA cases reported to SIVIGILA from 2016 to 2024, including categorization by SMA type, is illustrated in Figure 1. An overall upward trend in reported cases is evident, with the number of cases peaking in 2021. This trend may reflect factors such as increased awareness, enhanced reporting practices, or improvements in SMA diagnostic methods over time. SMA type 1 constitutes a substantial proportion of the total SMA cases reported annually, which likely reflects the severity of SMA type 1. The severity of SMA type 1 may lead to earlier diagnosis and more consistent reporting compared to milder SMA types​.

Annual frequency of clinical visits by SMA type

**Figure 2:**
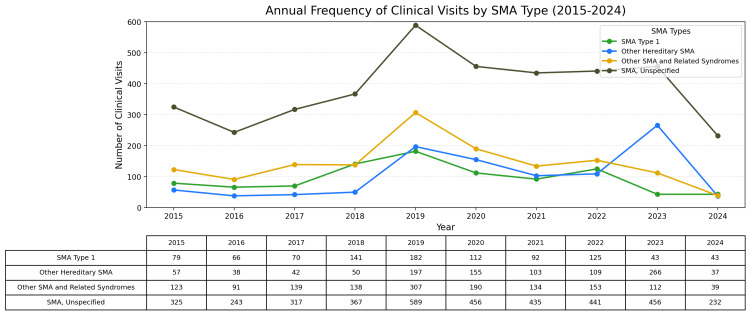
Annual frequency of healthcare encounters for patients with SMA types, from 2015 to 2024. This figure illustrates the higher frequency of clinical visits among SMA type 1 patients compared to other spinal muscular atrophy (SMA) types, reflecting the increased healthcare needs associated with the severe progression and symptoms of SMA type 1. These findings underscore the significant resource demands and the necessity for specialized support and interventions within the healthcare system to manage SMA type 1 effectively.

The annual frequency of clinical visits by SMA type is presented in Figure 2. SMA type 1 patients exhibited the highest frequency of clinical visits each year compared to other SMA types. This observation aligns with the increased healthcare needs of SMA type 1 patients, whose severe symptoms require frequent medical attention. The peak in clinical visits in 2019 may reflect both an increase in diagnosed cases and a greater demand for healthcare resources. This pattern underscores the need for resource allocation tailored to the more intensive care requirements of SMA type 1.

Sex distribution of SMA type 1 cases

**Figure 3:**
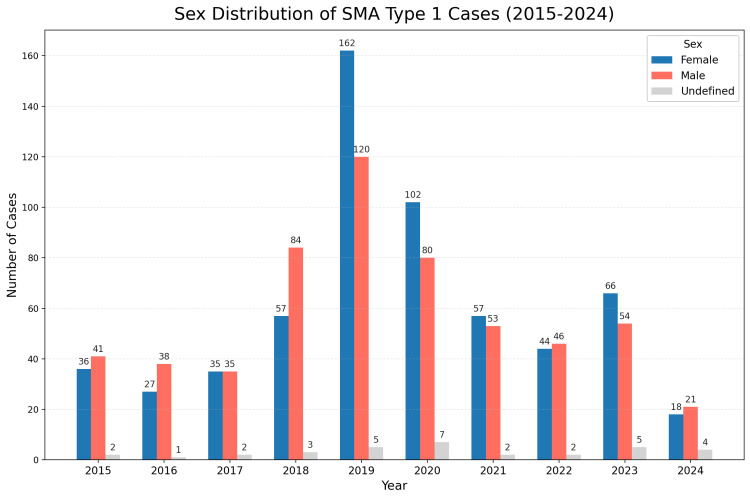
Sex distribution of SMA type 1 cases reported from 2015 to 2024. This figure illustrates the annual distribution of spinal muscular atrophy (SMA) type 1 cases by sex, with a generally balanced representation between male patients and female patients across the years. Although there are slight annual fluctuations, these variations do not suggest a significant sex-based trend in SMA type 1 prevalence. This indicates that sex does not appear to substantially influence the incidence or reporting of SMA type 1 in Colombia.

The sex distribution of SMA type 1 cases over the study period is illustrated in Figure 3. The analysis reveals a generally balanced representation between male patients and female patients across the years, though slight annual fluctuations are observed. These minor variations do not suggest a significant sex-based trend in SMA type 1 prevalence, indicating that sex does not appear to substantially influence the incidence or reporting of SMA type 1 in Colombia.

**Figure 4:**
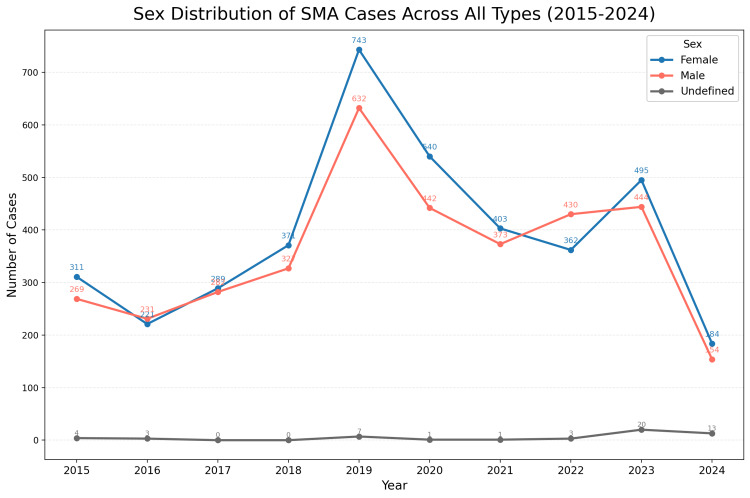
Sex distribution of SMA cases across all types from 2015 to 2024. This figure shows the yearly distribution of spinal muscular atrophy (SMA) cases by sex, including all SMA types. The data indicates a generally balanced representation between male patients and female patients over the study period, with some fluctuations each year. These findings suggest that sex does not significantly influence the occurrence or reporting trends of SMA cases in Colombia, supporting the conclusion that there is no notable sex-based bias in SMA prevalence.

Figure 4 provides the overall sex distribution across all SMA types, also showing a balanced representation between male patients and female patients, further supporting the conclusion that sex does not play a significant role in SMA occurrence or reporting trends in Colombia.

Geographic distribution of SMA type 1 cases

**Figure 5:**
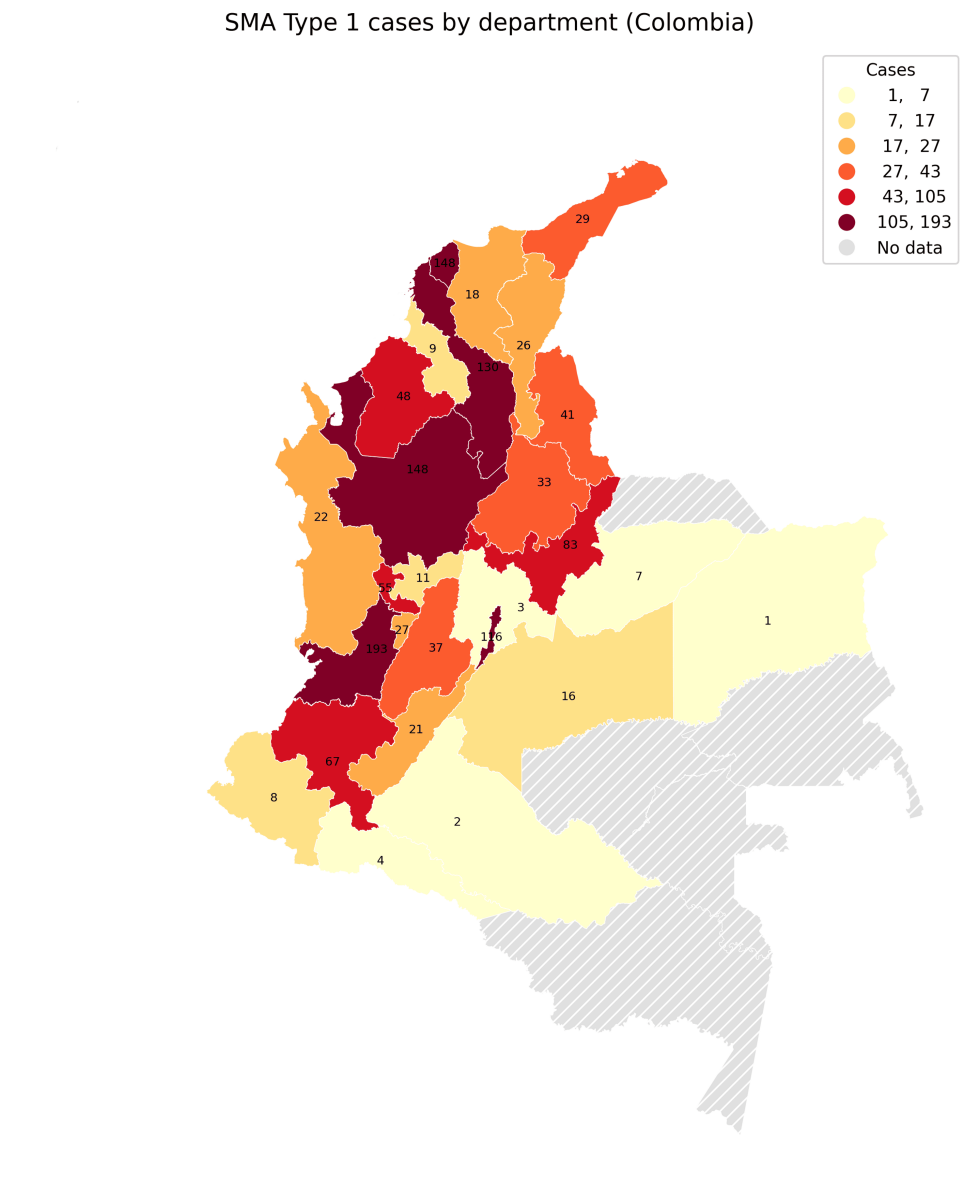
Geographic distribution of SMA type 1 cases by department in Colombia. This figure shows the concentration of cases across departments, with some areas exhibiting higher numbers. Departments such as Valle del Cauca, Atlántico, Antioquia, and Bogotá have notably high counts of spinal muscular atrophy (SMA) type 1 cases. These regional differences may reflect variations in healthcare access, genetic factors, or diagnostic practices across regions. This geographic variation highlights the need for targeted healthcare resources and awareness programs in departments with higher case numbers to enhance access to diagnosis and treatment for SMA patients.

Figure 5 presents the geographic distribution of SMA type 1 cases by department, revealing that certain departments exhibit higher concentrations of cases. These regional disparities may reflect differences in healthcare access, genetic predisposition, or diagnostic practices across departments. The geographic variation in SMA type 1 cases underscores the importance of targeted healthcare resources and awareness programs in regions with higher case concentrations, particularly to improve access to diagnostic and therapeutic support for SMA patients​.

## Discussion

This study provides a detailed overview of the epidemiology of SMA in Colombia, identifying significant trends and characteristics in the national SMA profile. Using data from the SIVIGILA and RIPS databases, we observed a marked peak in SMA incidence in 2019, followed by a plateau, likely reflecting changes in diagnostic accessibility and healthcare priorities during this period. SMA type 1, the most severe and resource-intensive form of the disease, constituted a significant proportion of cases, predominantly affecting young children who require extensive healthcare resources [22,23]. The geographic distribution of SMA incidence showed substantial variability, with higher concentrations in regions with more accessible healthcare facilities, underscoring possible disparities in SMA reporting and diagnosis across Colombia [4]. This study provides valuable insights into demographic patterns, clinical demands, and the regional distribution of SMA in Colombia, highlighting the healthcare burden associated with this rare condition.

Comparisons with international studies reveal both similarities and differences, particularly with data from regions sharing similar healthcare contexts. For example, Kimizu et al. in Japan and Jedrzejowska et al. in Poland observed a predominance of SMA type 1 cases, emphasizing the significant clinical severity and healthcare demand worldwide for this subtype [22,23]. However, while studies in Brazil and Cuba reported relatively stable SMA incidence over time, our data from Colombia showed a peak incidence in 2019, followed by a plateau, diverging from trends in other Latin American countries [4]. Nusinersen was the first disease-modifying drug approved in the world, initially by the U.S. Food and Drug Administration (FDA). In Colombia, the approval of nusinersen by the Instituto Nacional de Vigilancia de Medicamentos y Alimentos (INVIMA) in 2019 encouraged the nationwide implementation of different awareness-raising and training activities on SMA through congresses, conferences, and campaigns aimed at healthcare personnel, with the aim of promoting early diagnosis of SMA. This would explain why the incidence was significantly higher after 2019 compared to previous years. In addition, genetic studies are becoming easier to obtain than in previous years, which has favoured confirmation in an increasing number of patients, as reflected in the incidence of this disease.

Studies from high-income countries, such as that by Price et al. in Canada, documented consistent incidence rates over time, likely due to national newborn screening programs that allow earlier and standardized SMA detection [24]. In contrast, Colombia’s lack of a standardized genetic screening program may explain the observed temporal and geographic patterns with variability over time, as SMA diagnosis and reporting are heavily reliant on regional healthcare resources and provider awareness.

The sharp increase in SMA incidence in Colombia in 2019, followed by a plateau, may be attributed to several factors. The availability of more advanced genetic diagnostic tests likely improved diagnostic accuracy, enabling the identification and reporting of more cases and contributing to the incidence peak in 2019. This stabilization may reflect a new equilibrium, as previously undiagnosed cases were captured. Additionally, the onset of the COVID-19 pandemic in 2020 likely impacted SMA reporting, as healthcare priorities shifted and access to diagnostic and screening services was restricted. Similar impacts were noted in studies by Okamoto et al. in Japan and Burd et al. in the United States, suggesting that pandemic-related healthcare disruptions affected SMA detection and reporting [25,26]. Therefore, improved diagnostic capabilities before 2019 and pandemic-related disruptions may together explain the plateau in SMA incidence observed in Colombia after 2019.

The high incidence of SMA type 1 cases, along with the intensive clinical demands associated with this subtype, has significant implications for Colombia’s healthcare system, particularly in terms of resource allocation. SMA type 1 patients typically require extensive medical support, including respiratory care and physical therapy, as also noted in studies from Canada by Price et al. and Japan by Kimizu et al. [22,24]. In Colombia, these findings emphasize the need for targeted resource allocation to ensure an early, prompt diagnosis, genetic counseling, and specialized care programs, particularly in regions with limited healthcare access and higher SMA concentrations. Policies that expand regional access to SMA therapies and early intervention programs could improve disease management, reduce healthcare burdens, and improve patient outcomes. Additionally, establishing national guidelines for SMA management aligned with international standards could help standardize care and reduce disparities across Colombia’s diverse regions.

This study has limitations, mainly due to its reliance on secondary data from SIVIGILA and RIPS, which may result in underreporting, especially in rural or marginalized areas with limited access to healthcare. Furthermore, the lack of knowledge that still exists regarding this rare disease may also contribute to this underdiagnosis. Geographic disparities in access to healthcare may contribute to underdiagnosis in remote regions, as observed in the studies by Bouhouche et al. in Morocco and Verhaart et al., who reported similar challenges in diagnosing SMA in low-resource areas [4,27]. Furthermore, SMA cases with milder symptoms may remain undiagnosed, particularly in regions with limited access to specialized neurological care, potentially leading to a selection bias favoring cases from urban centers with better healthcare infrastructure. Alvarez et al. mention that children with SMA type 3b had been clinically diagnosed long before access to molecular genetic diagnosis was available [1]. These limitations may affect the generalizability of our findings, highlighting the need for regionally inclusive studies and better screening mechanisms for rare diseases such as SMA in Colombia, such as neonatal screening. This proposal has been raised in other publications, such as that of Kimizu et al., as a solution to the underdiagnosis presented in the population [18].

## Conclusions

This study provides valuable insights into the epidemiology of SMA in Colombia, highlighting trends, demographic patterns, and healthcare demands associated with the disease. An increase in incidence is described in 2019, with a subsequent trend towards stabilization. This increase in incidence is associated with a corresponding rise in healthcare encounters. Future research should prioritize detailed genetic studies to identify SMA subtypes specific to the Colombian population, in conjunction with long-term follow-up studies to monitor disease progression and healthcare outcomes. Enhanced rare disease surveillance systems, similar to those in high-income countries, could facilitate early detection and improve SMA tracking over time. Implementing a newborn screening program would be a pivotal step in promoting early diagnosis and timely intervention for SMA cases in Colombia. Continued research and policy development are essential to improving diagnostic accuracy, healthcare accessibility, and overall SMA management in Colombia, thus aligning with global standards and fostering a more equitable healthcare response for individuals affected by this rare disease.

## References

[1] Alvarez K, Suarez B, Palomino MA (2019). Observations from a nationwide vigilance program in medical care for spinal muscular atrophy patients in Chile. Arq Neuropsiquiatr.

[2] Franco-Toñánez C, Godoy-Sánchez L CG (2021). Clinical, epidemiological, and genetic characterization of patients with spinal muscular atrophy: series of 26 pediatric patients (Article in Spanish). Pediatria.

[3] D'Amico A, Mercuri E, Tiziano FD, Bertini E (2011). Spinal muscular atrophy. Orphanet J Rare Dis.

[4] Verhaart IE, Robertson A, Wilson IJ (2017). Prevalence, incidence and carrier frequency of 5q-linked spinal muscular atrophy - a literature review. Orphanet J Rare Dis.

[5] Angilletta I, Ferrante R, Giansante R (2023). Spinal muscular atrophy: an evolving scenario through new perspectives in diagnosis and advances in therapies. Int J Mol Sci.

[6] Messina S (2018). New directions for SMA therapy. J Clin Med.

[7] Bladen CL, Thompson R, Jackson JM (2014). Mapping the differences in care for 5,000 spinal muscular atrophy patients, a survey of 24 national registries in North America, Australasia and Europe. J Neurol.

[8] Mercuri E, Finkel RS, Muntoni F (2018). Diagnosis and management of spinal muscular atrophy: part 1: recommendations for diagnosis, rehabilitation, orthopedic and nutritional care. Neuromuscul Disord.

[9] Gil-Rojas Y, Suárez-Obando F, Amaya-Granados D, Prieto-Pinto L, Samacá-Samacá D, Ortiz B, Hernández F (2023). Burden of disease of spinal muscular atrophy linked to chromosome 5q (5q-SMA) in Colombia. Expert Rev Pharmacoecon Outcomes Res.

[10] Tan H, Gu T, Chen E, Punekar R, Shieh PB (2019). Healthcare utilization, costs of care, and mortality among patients with spinal muscular atrophy. J Health Econ Outcomes Res.

[11] Finkel RS, Chiriboga CA, Vajsar J (2016). Treatment of infantile-onset spinal muscular atrophy with nusinersen: a phase 2, open-label, dose-escalation study. Lancet.

[12] Wohnrade C, Velling AK, Mix L (2023). Health-related quality of life in spinal muscular atrophy patients and their caregivers-a prospective, cross-sectional, multi-center analysis. Brain Sci.

[13] Wang CH, Finkel RS, Bertini ES (2007). Consensus statement for standard of care in spinal muscular atrophy. J Child Neurol.

[14] Groen EJ, Talbot K, Gillingwater TH (2018). Advances in therapy for spinal muscular atrophy: promises and challenges. Nat Rev Neurol.

[15] Finkel RS, Mercuri E, Meyer OH (2018). Diagnosis and management of spinal muscular atrophy: part 2: pulmonary and acute care; medications, supplements and immunizations; other organ systems; and ethics. Neuromuscul Disord.

[16] (2017). Vital Statistics: Provisional Figures.

[17] Oliveira Netto AB, Brusius-Facchin AC, Lemos JF (2023). Neonatal screening for spinal muscular atrophy: a pilot study in Brazil. Genet Mol Biol.

[18] Ito M, Yamauchi A, Urano M, Kato T, Matsuo M, Nakashima K, Saito K (2022). Epidemiological investigation of spinal muscular atrophy in Japan. Brain Dev.

[19] Medrano S, Monges S, Gravina LP (2016). Genotype-phenotype correlation of SMN locus genes in spinal muscular atrophy children from Argentina. Eur J Paediatr Neurol.

[20] Febrer A, Vigo M, Fagoaga J, Medina-Cantillo J, Rodríguez N, Tizzano E (2011). Hammersmith functional rating scale for children with spinal muscular atrophy. Validation of the Spanish version (Article in Spanish). Rev Neurol.

[21] Butterfield RJ (2021). Spinal muscular atrophy treatments, newborn screening, and the creation of a neurogenetics urgency. Semin Pediatr Neurol.

[22] Kimizu T, Ida S, Okamoto K (2021). Spinal muscular atrophy: diagnosis, incidence, and newborn screening in Japan. Int J Neonatal Screen.

[23] Jedrzejowska M, Milewski M, Zimowski J (2010). Incidence of spinal muscular atrophy in Poland--more frequent than predicted?. Neuroepidemiology.

[24] Price TR, Hodgkinson V, Westbury G (2024). A study on the incidence and prevalence of 5q spinal muscular atrophy in Canada using multiple data sources. Can J Neurol Sci.

[25] Okamoto K, Nishio H, Motoki T (2022). Changes in the incidence of infantile spinal muscular atrophy in Shikoku, Japan between 2011 and 2020. Int J Neonatal Screen.

[26] Burd L, Short SK, Martsolf JT, Nelson RA (1991). Prevalence of type I spinal muscular atrophy in North Dakota. Am J Med Genet.

[27] Bouhouche A, Benomar A, Birouk N, Bouslam N, Ouazzani R, Yahyaoui M, Chkili T (2003). High incidence of SMN1 gene deletion in Moroccan adult-onset spinal muscular atrophy patients. J Neurol.

